# Predicting Survival Status in COVID-19 Patients: Machine Learning Models Development with Ventilator-Related and Biochemical Parameters from Early Stages: A Pilot Study

**DOI:** 10.3390/jcm13206190

**Published:** 2024-10-17

**Authors:** Shin-Ho Chou, Cheng-Yu Tsai, Wen-Hua Hsu, Chi-Li Chung, Hsin-Yu Li, Zhihe Chen, Rachel Chien, Wun-Hao Cheng

**Affiliations:** 1 Respiratory Therapy, Department of Pulmonary Medicine, Taipei Medical University Hospital, Taipei 110, Taiwan; 164074@h.tmu.edu.tw (S.-H.C.); b117101018@tmu.edu.tw (C.-Y.T.); clchung@tmu.edu.tw (C.-L.C.); 886004@h.tmu.edu.tw (H.-Y.L.); 2 Division of Pulmonary Medicine, Department of Internal Medicine, Shuang Ho Hospital, Taipei Medical University, New Taipei City 235, Taiwan; 3 School of Respiratory Therapy, College of Medicine, Taipei Medical University, Taipei 110, Taiwan; 4 School of Biomedical Engineering, College of Biomedical Engineering, Taipei Medical University, Taipei 110, Taiwan; 5 Department of Internal Medicine, School of Medicine, College of Medicine, Taipei Medical University, Taipei 110, Taiwan; b117105061@tmu.edu.tw; 6 Department of Civil and Environmental Engineering, Imperial College London, London SW7 2AZ, UK; fc323@ic.ac.uk; 7 Graduate Institute of Clinical Medicine, College of Medicine, Taipei Medical University, Taipei 100, Taiwan; r09850011@ntu.edu.tw; 8 Respiratory Therapy, Division of Pulmonary Medicine, Department of Internal Medicine, Wan Fang Hospital, Taipei Medical University, Taipei 110, Taiwan

**Keywords:** COVID-19, random forest, C-Reactive Protein (CRP), ventilator weaning, survival prediction

## Abstract

**Objective**: Coronavirus disease 2019 (COVID-19) can cause intubation and ventilatory support due to respiratory failure, and extubation failure increases mortality risk. This study, therefore, aimed to explore the feasibility of using specific biochemical and ventilator parameters to predict survival status among COVID-19 patients by using machine learning. **Methods**: This study included COVID-19 patients from Taipei Medical University-affiliated hospitals from May 2021 to May 2022. Sequential data on specific biochemical and ventilator parameters from days 0–2, 3–5, and 6–7 were analyzed to explore differences between the surviving (successfully weaned off the ventilator) and non-surviving groups. These data were further used to establish separate survival prediction models using random forest (RF). **Results**: The surviving group exhibited significantly lower mean C-reactive protein (CRP) levels and mean potential of hydrogen ions levels (pH) levels on days 0–2 compared to the non-surviving group (CRP: non-surviving group: 13.16 ± 5.15 ng/mL, surviving group: 10.23 ± 5.15 ng/mL; pH: non-surviving group: 7.32 ± 0.07, survival group: 7.37 ± 0.07). Regarding the survival prediction performanace, the RF model trained solely with data from days 0–2 outperformed models trained with data from days 3–5 and 6–7. Subsequently, CRP, the partial pressure of carbon dioxide in arterial blood (PaCO_2_), pH, and the arterial oxygen partial pressure to fractional inspired oxygen (P/F) ratio served as primary indicators in survival prediction in the day 0–2 model. **Conclusions**: The present developed models confirmed that early biochemical and ventilatory parameters—specifically, CRP levels, pH, PaCO_2_, and P/F ratio—were key predictors of survival for COVID-19 patients. Assessed during the initial two days, these indicators effectively predicted the likelihood of successful weaning of from ventilators, emphasizing their importance in early management and improved outcomes in COVID-19-related respiratory failure.

## 1. Introduction

Coronavirus disease 2019 (COVID-19), caused by the new coronavirus named severe acute respiratory syndrome coronavirus, rapidly spread across the globe, posing a significant challenge to global public health. It resulted in the widespread transmission of atypical viral pneumonia [1]. The first infection was reported at the end of December 2019. Almost 3 years later, the World Health Organization had recorded over 768 million confirmed COVID-19 cases, with 6.9 million deaths [2]. A multi-center epidemiological study revealed that approximately 76% of COVID-19 patients required supplemental oxygen, and around 9% of these patients needed tracheal intubation and invasive mechanical ventilation [3]. Moreover, approximately one-third of patients were unable to be successfully extubated, leading to an increased mortality rate [1]. Therefore, further investigation is still required to refine predictors of survival with successful extubation.

To assess COVID-19 progression, serum biomarkers in severe patients have been widely used and can potentially predict outcomes. For instance, C-reactive protein (CRP) is an acute inflammatory protein that increases during an early-phase response to inflammation [4]. Elevated white blood cell (WBC) counts often signal inflammation and the immune response to infection [5]. Additionally, ferritin, a clinical marker of iron storage, induces macrophage accumulation and enhances the production of reactive oxygen species during inflammation [6]. Elevated levels of CRP, WBC, and ferritin were linked to mortality in various diseases, including COVID-19 [7,8,9,10,11]. Also, the severity of COVID-19 is associated with elevated inflammatory markers, such as increased WBC counts, elevated CRP and ferritin levels, leukocytosis, neutrophilia, elevated D-dimer and procalcitonin, as well as CT findings of bilateral patchy shadows or ground glass opacity [6,12,13]. Moreover, the use of invasive mechanical ventilation in COVID-19 patients is significantly associated with increased mortality [14]. However, the potential of these inflammatory profiles to serve as predictors of successful extubation remains unknown. Further research is needed to develop models that confirm whether biochemical and ventilatory parameters are predictors of survival for COVID-19 patients.

Arterial blood gas (ABG) variable parameters, including pH, partial pressure of oxygen (PaO_2_), and partial pressure of carbon dioxide (PaCO_2_), as well as the mean airway pressure (MAP and ratio of arterial oxygen partial pressure to fractional inspired oxygen (P/F ratio) are commonly used to assess the oxygenation status and severity of patients with acute hypoxemic respiratory failure who require invasive ventilator support [15]. Nevertheless, additional research is needed to determine whether ABG measurements and ventilator-related parameters can serve as reliable predictors of ventilator weaning.

This retrospective study collected inflammatory profiles, ABG measurements, and ventilator parameters during the first week following the confirmation of a COVID-19 diagnosis. The aim of this study was to investigate the associations of these variables with the outcomes of COVID-19 patients on ventilators and to further employ them to predict distinct clinical outcomes regarding survival status (i.e., survival and successful weaning of from the ventilator versus non-survival). This study compared these parameters during the first 3 days (days 0–2), data from the subsequent period (days 3–5), and the remaining days (days 6–7). Additionally, we utilized a machine learning approach, random forest (RF) model, to compare predictive capabilities among the day 0–2, 3–5, and 6–7 periods. The novelty of this study lies in its methodological approach, utilizing a machine learning model—RF—to uniquely compare and predict survival outcomes based on early vs. later measurements, which has not been extensively explored in the current literature. The proposed model could provide new insights into the timing and effectiveness of interventions for COVID-19 patients on ventilators.

## 2. Materials and Methods

### 2.1. Ethics

The present study retrospectively obtained datasets from both the Taipei Medical University (TMU) Hospital (Taipei City, Taiwan) and Taipei Medical University-Shuang Ho Hospital (New Taipei City, Taiwan). The protocol for data collection, preservation, de-identification, and application of statistical approaches was reviewed and approved by the Joint Institutional Review Board of TMU (TMU-JIRB: N202209038 and TMU-JIRB: N202303023). All implemented procedures adhered to the approved guidelines.

### 2.2. Data Acquisition Procedures

Preliminary data were obtained from patients diagnosed with COVID-19 who had a cycle threshold (Ct) value of less than 40, had not received vaccination, were admitted to the intensive care unit (ICU) of the pulmonary medicine department, and were placed on ventilator support between May 2021 and May 2022. Data were further selected based on the following criteria: (1) patients aged 20 years or older, (2) biochemical data and ABG measurements sampled from patients every 1 to 2 days, (3) detailed records of the daily ventilator settings from patients being accessible, (4) patients administered immune reaction inhibitors (e.g., tocilizumab) and antiviral medicine (e.g., remdesivir) upon confirmation of a COVID-19 diagnosis, and (5) patients who were hospitalized in an ICU for a minimum of 1 week (days ≥ 7). Data were collected from patients meeting these criteria, including background details, biochemical and ABG data, and relevant ventilator settings. Patients were excluded if their case information is incomplete, or if they were admitted without relevant clinical examinations and subsequently weaned from the ventilator. Subsequently, per the final clinical outcome, data were divided into two groups, namely surviving (successfully weaned off the ventilator) or non-surviving groups, for further comparison.

### 2.3. Demographics, Biochemical Data, ABG Measurements, and Ventilator-Associated Parameters

Concerning data types, this study acquired demographic characteristics, lifestyle habits, and confirmed comorbidities from historical medical records of eligible patients. The Charlson Comorbidity Index (CCI) was calculated for each patient based on the retrieved data. Comorbidity severity was then classified into the following three categories: mild (CCI scores of 1–2), moderate (CCI scores of 3–4), and severe (CCI scores ≥ 5) [16,17]. For biochemical data, considering patients in the ICU regularly underwent biochemical measurements to assess COVID-19 progression and treatment efficiency, this study obtained concentrations of CRP and ferritin, and WBC counts from patients during the first week after initiating mechanical ventilation. Next, as part of standard care, ABG measurements were measured daily to assess the level of support needed from mechanical ventilation. Therefore, this study included these ABG measurements in the dataset, specifically the value of potential of hydrogen (pH), bicarbonate (HCO_3_^−^) concentrations, and oxygen levels (PaO_2_) and carbon dioxide levels (PaCO_2_) in arterial blood. Furthermore, relevant ventilator-associated parameters, such as P/F ratio and MAP, were also collected. All retrieved data were utilized for further analysis.

### 2.4. Statistical Analysis

All statistical analyses were carried out using the open-source Python library, Scikit-learn (version 0.21.2; Python Software Foundation, Fredericksburg, VA, USA). For continuous variables, this study first performed the Shapiro–Wilk test to assess the distributional assumptions of each variable. For normally distributed variables, Student’s *t*-test was utilized, while the Mann–Whitney U-test was employed for non-normally distributed variables. For categorical variables (e.g., comorbidities and tobacco usage), the study implemented a Chi-squared test to discern differences between survivors and non-survivors. To investigate relationships of changes in biochemical data, ABG measurements, and ventilator-associated parameters with survival status, this study employed logistic regressions to examine relationships between the surviving and non-surviving patient groups. Specifically, the following two types of logistic regression models were employed: crude models (without adjustment for confounding factors) and multivariable logistic regression models with adjustments for age, sex, body mass index (BMI), and CCI. Results of these regression models are presented as odds ratios (ORs) with 95% confidence intervals (CIs). The statistical significance was accepted at *p* < 0.05.

### 2.5. Survival Status Prediction and Feature Importance

This study further employed ensemble methods, specifically random forest (RF) models, to utilize variations in biochemical data, ABG measurements, and ventilator parameters across different time spans for predicting survival status. This approach has previously been used to predict the risk of COVID-19 progression and to compare the importance of input features across different spans [18,19]. Therefore, this study employed RF to assess impacts of the aforementioned parameters on survival status, comparing data from different periods (means of days 0–2, 3–5, and 6–7).

Regarding the details of model establishment and feature importance, the procedure for developing and interpreting the model was as follows. Initially, to ensure model stability and time efficiency, the fundamental decision-making unit of the RF model, the classification and regression tree, was set to 250 [20]. Afterwards, three distinct model categories were formulated using data from the entire week. These categories included models that solely considered data from the first 3 days (days 0–2), data from the following period (days 3–5), and data from the remaining days (days 6–7). The amassed data were then iteratively introduced during the training phase to develop the RF model. The performance of the established models was evaluated based on several indicators, including equal error rates (ERRs, the point at which both false positive and false negative rates are equal), accuracy, F1 scores (the harmonic averages of precision and recall), and the area under the receiver operating characteristic curve (AUROC). Additionally, the feature importance of input variable within the RF was determined using the mean decrease in accuracy technique, with results visually represented through a bar chart. To elaborate, the mean decrease in accuracy is a method that computes feature importance based on the mean reduction in accuracy when values of a feature are randomly shuffled among samples [21].

## 3. Results

### 3.1. Demographics of Recruited Participants

The retrieved dataset included 56 individuals, divided into two groups based on clinical outcomes (Table 1). The surviving group consisted of 29 patients, while the non-surviving group consisted of 27 patients. Both groups were predominantly composed of males, and the mean age, CCI, and number of hospital days of the surviving group were lower than those of the non-surviving group, although the differences were not statistically significant. In terms of tobacco use status, the majority in both groups were non-smokers (over 80%).

### 3.2. Comparisons of Biochemical Measurements Between the Surviving and Non-Surviving Groups

Table 2 presents a comparison of mean values of biochemical measurements between the surviving and non-surviving groups. It was observed that patients in the surviving group had significantly higher mean CRP values during the initial 3-day period (days 0–2) in contrast to the non-surviving group (survival group: 12.62 ± 8.15 mg/L, non-surviving group: 7.51 ± 6.34 mg/L, *p* < 0.05). However, mean CRP values during the subsequent periods (days 3–5 and 6–7) exhibited no significant disparities between the groups. Regarding WBC counts, the non-surviving group exhibited a steady rise in mean values, showcasing a significant difference compared to mean values of surviving group during days 3–5 (surviving group: 10.23 ± 5.15 × 10^3^/μL, non-surviving group: 13.16 ± 5.15 × 10^3^/μL, *p* < 0.05) and days 6–7 (surviving group: 10.55 ± 4.52 × 10^3^/μL, non-surviving group: 13.63 ± 6.31 × 10^3^/μL, *p* < 0.05).

### 3.3. Comparisons of ABG Measurements and Ventilator-Associated Parameters Between the Surviving and Non-Surviving Groups

The ABG measurements and ventilator-associated parameters for the two groups are summarized and compared in (Table 3). Patients in the surviving group exhibited significantly higher mean values of pH within the normal range during the initial 3-day period (days 0–2), compared to the non-surviving group (survival group: 7.37 ± 0.07, non-surviving group: 7.32 ± 0.07, *p* < 0.05). For the P/F ratio, which indicates respiratory efficiency, the surviving group exhibited a gradual increase in mean values over time, ranging from 245.09 to 298.97, and showed a significant difference compared to the non-surviving group during days 6–7 (surviving group: 298.97 ± 158.06, non-surviving group: 190.82 ± 93.9, *p* < 0.05).

### 3.4. Associations of Biochemical Data, ABG Measurements, and Ventilator-Associated Parameters with Survivability

The OR values, representing the impact of biochemical data, ABG measurements, and ventilator-associated parameters on survival, are displayed in (Table 4 and Table 5). An increase of 1 unit in the mean CRP of days 0–2 in both the crude model and the model adjusted for age, sex, BMI, and CCI was significantly associated with increased ORs of survival (crude model: OR: 1.1, 95% CI: 1.02–1.21, *p* < 0.05; adjusted model: OR: 1.11, 95% CI: 1.01–1.22, *p* < 0.05). Similarly, for pH values on days 0–2, a 1-unit increase in this variable was significantly associated with increased ORs of survival in both the crude and adjusted models (crude model: OR: 2.08, 95% CI: 1.11–3.89, *p* < 0.05; adjusted model: OR: 2.22, 95% CI: 1.08–4.59, *p* < 0.05). Regarding blood gas pressure, in the adjusted models, 1-unit increases in the PaCO_2_ mean of days 0–2 and days 6–7 were significantly associated with decreased ORs of survival (mean of days 0–2: OR: 0.91, 95% CI: 0.84–0.99, *p* < 0.05; mean of days 6–7: OR: 0.92, 95% CI: 0.85–0.99, *p* < 0.05). In contrast, 1-unit increases in the PaO_2_ mean of days 3–5 and days 6–7 were significantly associated with elevated ORs of survival in the adjusted models (mean of days 3–5: OR: 2.32, 95% CI: 1.11–4.86, *p* < 0.05; mean of days 6–7: OR: 2.11, 95% CI: 1.06–4.21, *p* < 0.05). In terms of ventilator-associated parameters, 1-unit increases in the P/F ratio over time were all significantly associated with increased ORs of survival in the adjusted models (mean of days 0–2: OR: 2.24, 95% CI: 1.02–4.93, *p* < 0.05; mean of days 3–5: OR: 2.91, 95% CI: 1.23–6.89, *p* < 0.05; mean of days 6–7: OR: 3.84, 95% CI: 1.54–9.53, *p* < 0.05). On the other hand, a 1-unit increase in the MAP of days 0–2 was significantly associated with a decreased OR of survival in the adjusted model (OR: 0.49, 95% CI: 0.24–0.99, *p* < 0.05).

### 3.5. Feature Importance Impacting Survival Status

**Figure 1:**
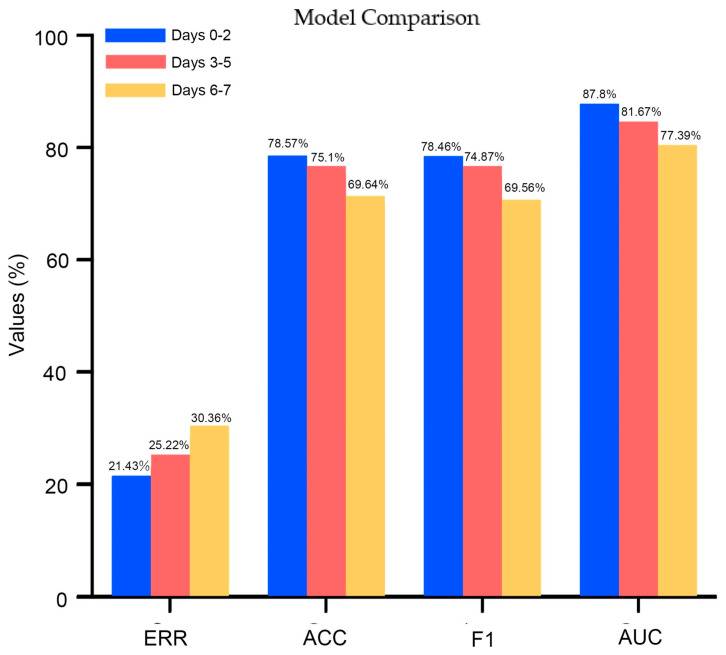
Illustrates the performance metrics of models trained with the following three different periods: Days 0–2, Days 3–5, and Days 6–7. The metrics include Error Rates (ERRs), Accuracy, F1 Scores, and Area Under the Receiver Operating Characteristic Curve (AUROC).

**Figure 2:**
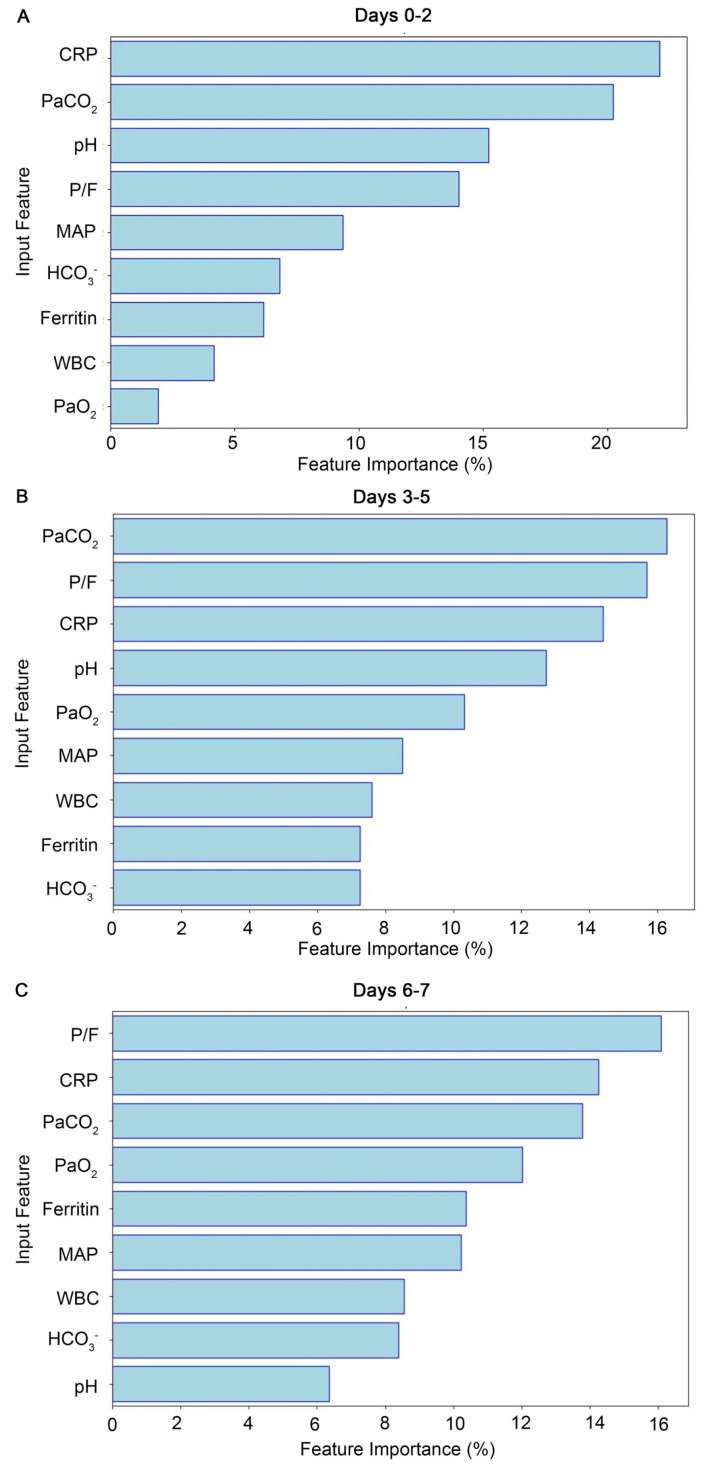
Shows the feature importance for predicting survival (with successful weaning) versus non-survival using data from Days (**A**) 0–2, (**B**) 3–5, and (**C**) 6–7. During days 0–2, CRP is the most important feature, followed by PaCO_2_, pH, and P/F ratio.

Metrics of the model performance, including ERRs, accuracy, F1 scores, and the AUROC for the models trained with data from the three periods, are illustrated in (Figure 1). The model trained on data from days 0–2 relatively outperformed the other models trained on data from days 3–5 and 6–7, showing higher accuracy, F1 scores, and AUROC, but a lower ERR for survival prediction. In terms of feature importance, for the initial 3-day period (days 0–2), the level of CRP demonstrated the highest importance for clinical outcomes, followed by PaCO_2_, pH, and the P/F ratio (Figure 2).

### 3.6. Supplementary Analysis: Comparative Assessment of Biochemical Data, ABG Measurements, and Ventilator-Associated Parameters at Different Time Intervals Between the Surviving and Non-Surviving Groups

This study conducted additional analyses to calculate mean values of biochemical data, ABG measurements, and ventilator-associated parameters at different time intervals (i.e., days 0–2, 0–5, and 0–7). These analyses aimed to compare short-term variations and long-term trends between the two groups, and the results of these analyses are presented in (Tables S1 and S2). Surviving patients had significantly higher mean CRP values at the 3-day interval (days 0–2, surviving group: 12.62 ± 8.15 mg/L, non-surviving group: 7.51 ± 6.34 mg/L, *p* < 0.05) and 5-day interval (days 0–5, surviving group: 9.34 ± 5.85 mg/L, non-surviving group: 6.01 ± 4.89 mg/L, *p* < 0.05). For ABG details and ventilator-associated parameters, the surviving group exhibited significantly higher mean values of pH within the normal range at all intervals (days 0–2, surviving group: 7.37 ± 0.07, non-surviving group: 7.32 ± 0.07, *p* < 0.05; days 0–5, surviving group: 7.39 ± 0.09, non-surviving group: 7.35 ± 0.06, *p* < 0.05; days 0–7, surviving group: 7.39 ± 0.05, non-surviving group: 7.36 ± 0.06, *p* < 0.05). For the respiratory efficiency indicator of the P/F ratio, the surviving group showed higher mean values compared to the non-surviving group, and a significant difference was observed at the mean of days 0–7 (surviving group: 273.34 ± 136.68, non-surviving group: 203.5 ± 78.07, *p* < 0.05).

### 3.7. Supplementary Analysis: Feature Importance Impacting Survival Status Using Data at Different Time Intervals

This study further developed RF models using data from various time intervals to compare the effects on clinical outcomes (i.e., survival versus non-survival) by considering short-term variations and long-term trends (Figure S1) demonstrates the feature importance distributions of the 5-day and 7-day mean models. Similar to the results of the 3-day mean model (i.e., the model trained using data from days 0–2), PaCO_2_ and CRP levels were the first and third most important features, respectively, in the 5-day mean model, with pH being the second most important feature. Regarding the 7-day mean model, the P/F ratio, pH, and the CRP level were the three most important features. Subsequently, this study also evaluated the performance of the established models, as presented in (Figure S2). Similarly, the model trained on data from days 0–2 (3-day mean model) exhibited the highest accuracy, F1 scores, and AUROC, but a lower ERR for survival prediction compared to the 5-day and 7-day mean models. In other words, the model trained using the short-term data (days 0–2) had superior predictive performance compared to models trained using long-term data (i.e., day 0–5 and 0–7 data).

## 4. Discussion

This study investigated associations and ORs between these factors in the surviving and non-surviving groups, specifically in three different periods (days 0–2, 3–5, and 6–7). Additionally, in predicting survival with successful weaning or mortality, the RF model, considering the day 0–2 period, demonstrated higher accuracy and F1 scores, along with a lower EER, compared to the model incorporating data from other periods.

The surviving group exhibited significantly higher CRP levels in the day 0–2 period and had lower WBC counts in the day 3–5 and 6–7 periods compared to the non-surviving group. Moreover, the possibility of surviving was positively associated with CRP levels during the day 0–2 period (ORs > 1). A previous study indicated that increased CRP levels were correlated with the severity of disease. However, it is important to note that elevated CRP levels can sometimes play a protective role against infection by reflecting innate immune activation in response to foreign pathogens. This process involves the following three major effector functions: complement activation, opsonization, and the induction of phagocytosis [17]. This elevation reflects a protective role in the initial stages of COVID-19 infection [4,22]. Additionally, it was found that elevated CRP levels are associated with an increased risk of mortality in COVID-19 patients, with an optimal cutoff value of ≥40 mg/L [7]. In the present study, the surviving group exhibited a relatively higher CRP level of 12.62 mg/L during the day 0–2 period compared to the non-surviving group. These findings suggest that maintaining a moderate range of CRP levels may be reasonable for an effective inflammatory response during early stages of the disease.

Effective management of acute respiratory failure requires maintaining adequate oxygenation and pH balance [23]. Previous researchers indicated that various parameters, including the P/F ratio, PaO_2_, PaCO_2_, and alveolar–arterial gradient, can serve as predictors of 28-day mortality in patients with COVID-19 [24]. Based on these outcomes, an increased possibility of surviving was associated with arterial pH levels during the day 0–2 period, PaO_2_ from the day 3–5 to day 6–7 period, and P/F ratio from the day 0–2 to day 6–7 period (ORs >1). Conversely, the possibility of surviving was negatively associated with PaCO_2_ levels during the day 0–2 and day 6–7 periods (ORs < 1). These findings suggest that lower arterial pH levels and higher PaCO_2_ levels, indicative of respiratory acidosis, can pose challenges during the weaning process, potentially leading to a higher probability of failed extubation in early stages of the disease. Additionally, the oxygen status (PaO_2_ and P/F ratio) was found to be an important predictor of survival with successful weaning or non-survival across the day 3–5 to day 6–7 periods. In terms of ventilator-associated parameters, MAP is a critical indicator for patients receiving invasive ventilator support, as it reflects both the work of breathing supported by the ventilator and the severity of the disease [25]. Previous studies demonstrated that MAP reflects both circulatory and pulmonary impairment during mechanical ventilation, both of which contribute to poorer outcomes [25,26]. In these observations, an increased MAP during the day 0–2 period was associated with decreased survival rates, suggesting that low MAP is crucial in predicting survival with successful weaning or non-survival.

Regarding model performance based on data from different time periods, the current study revealed that the RF model performed best during the day 0–2 period compared to the day 3 to 5 and day 6 to 7 periods, even when considering the collective day 0 to 5 and day 0 to 7 periods collectively. This finding suggests that the first three days of ventilator use are more informative for predicting outcomes of COVID-19 patients. These results are consistent with a previous study which indicated that prolonged use of ventilators is associated with an increased risk of complications, worsened outcomes, and a higher impact on mortality rates of COVID-19 patients [27]. We also conducted an analysis of feature importance to identify primary parameters and biochemical data that contributed to the predictive ability of the model. Among these parameters, CRP demonstrated higher feature importance in the day 0–2 and day 0–5 periods. This finding indicates that CRP levels during the initial phase of ventilator use may play a crucial role in predicting patient outcomes. These findings align with the important role of CRP mentioned above in activating the innate immune response against the COVID-19 virus during early stages of the disease [4,22].

COVID-19 is associated with many comorbidities, with the most common being hypertension (56.6%), obesity (41.7%), and diabetes (33.8%) [28]. A significant increase in mortality was observed among COVID-19-infected individuals with these comorbidities, particularly in male patients aged ≥50 years [29]. In this study, the CCI score was not over 1, and no significant difference was found between the survival and non-survival groups, suggesting the impact of comorbidities was not as pronounced. Moreover, multiple new variants of COVID-19, such as Alpha (B.1.1.7), Beta (B.1.351), Gamma (P.1), Delta (B.1.617.2), and Omicron (B.1.1.529), have been associated with increased transmissibility and virulence [30]. A previous study indicated that elderly patients with comorbidities face the worst outcomes with Omicron infections, characterized by longer hospitalizations and higher mortality rates [31]. However, further investigation is required to explore the correlation between comorbidities and the impact of different COVID-19 variants.

The present study has several limitations that should be acknowledged and addressed in future research. First, the retrospective nature of data collection limited the availability of daily measurements, and intermittent sampling may have restricted our ability to capture the full dynamics of certain parameters. Second, potential impacts of specific comorbidities on outcomes were not thoroughly investigated, warranting further exploration. Additionally, while (Table 1) shows no significant differences in demographic characteristics and comorbidities between the surviving and non-surviving groups, other factors such as disease severity and treatment interventions should be considered in future analyses. Third, due to the small sample size, the conclusions drawn from this study may not be adequately powered. Increasing the sample size and including data from multiple centers would strengthen future research. Finally, the absence of compliance and resistance data in ventilated patients highlights a need for more comprehensive evaluations of respiratory disease severity [25].

## 5. Conclusions

The findings indicated that the surviving group was significantly associated with increased levels of CRP, pH, PaO_2_, and the P/F ratio. Additionally, machine learning models, namely RF, were developed using early-stage biochemical and ventilator-associated parameters to predict survival with successful weaning and mortality in COVID-19 patients. The results revealed that data from the day 0–2 period exhibited higher precision, accuracy, and F1 scores, as well as a lower ERR, compared to models using data from other time periods. These results suggested that an appropriate inflammatory response, adequate oxygenation, and pH balance are crucial factors for a higher probability of successful ventilator weaning. Furthermore, the day 0–2 period emerged as having a greater impact on survival outcomes compared to other time periods. Altogether, the current established machine learning models confirmed that early biochemical and ventilatory markers can predict survival in COVID-19 patients, highlighting the value of these markers for early assessment and management.

## Figures and Tables

**Table 1:** Comparisons of demographic characteristics and comorbidity details of subjects stratified by survival status.

Categorical Variables	Survival Group (*n* = 29)	Non-Survival Group (*n* = 27)	*p*
Age (year) ^a^	69.17 ± 10.6	73.59 ± 7.82	0.08
Sex (male/female) ^b^	22/7	19/12	0.23
Body mass index (kg/m^2^) ^c^	25.43 ± 4.5	27.58 ± 8.48	0.47
GCS (score) ^c^	12.17 ± 4.28	12.15 ± 4.61	0.99
CCI (score) ^c^	0.52 ± 0.63	0.81 ± 0.88	0.23
Hospital day (*n*) ^c^	18.31 ± 15.36	19.85 ± 13.57	0.33
Comorbidities (*n*, %) ^b^			
Cardiovascular diseases	8 (27.59%)	4 (14.81%)	0.24
Hypertension	13 (44.83%)	18 (66.67%)	0.11
Chronic heart failure	0 (0.0%)	1 (3.7%)	0.97
COPD	1 (3.45%)	1 (3.7%)	0.51
Asthma	0 (0.0%)	2 (7.41%)	0.44
Diabetes mellitus	9 (31.03%)	10 (37.04%)	0.64
Acute kidney injury	8 (27.59%)	12 (44.44%)	0.19
Dementia	1 (3.45%)	2 (7.41%)	0.95
Tobacco usage (*n*, %) ^b^			0.17
Current smoker	1 (3.45%)	5 (18.52%)	
Non-smoker	28 (96.55%)	22 (81.48%)	

Abbreviations: GCS, Glasgow Coma Scale; CCI, Charlson comorbidity index; COPD, Chronic obstructive pulmonary disease. Data are expressed as the mean ± standard deviation. ^a^ Differences between groups were determined by Student’s *t*-test. ^b^ Differences between groups were determined by Chi-squared test. ^c^ Differences between groups were determined by Mann–Whitney U-test. The *p*-values were measured by comparing the survival and non-survival groups.

**Table 2:** Comparisons of biochemistry measurements of subjects stratified by survival status.

Categorical Variables	Survival Group (*n* = 29)	Non-Survival Group (*n* = 27)	*p*
CRP (mg/L)			
Mean of Days 0–2	12.62 ± 8.15	7.51 ± 6.34	<0.05
Mean of Days 3–5	6.06 ± 6.3	4.51 ± 6.23	0.29
Mean of Days 6–7	3.27 ± 4.78	4.91 ± 6.23	0.53
Ferritin (ng/mL)			
Mean of Days 0–2	1370.4 ± 864.34	3701.1 ± 12137.88	0.39
Mean of Days 3–5	962.19 ± 582.5	2420.93 ± 7551.5	0.58
Mean of Days 6–7	784.98 ± 438.32	1254.21 ± 2167.77	0.34
WBC (10^3^/μL)			
Mean of Days 0–2	10.24 ± 5.47	11.39 ± 4.9	0.34
Mean of Days 3–5	10.23 ± 5.15	13.16 ± 5.15	<0.05
Mean of Days 6–7	10.55 ± 4.52	13.63 ± 6.31	<0.05

Abbreviations: CRP, C-reactive protein; WBC, White blood cell count. Data are expressed as the mean ± standard deviation. Differences between groups were determined by Mann–Whitney U-test. The *p*-values were measured by comparing the survival and non-survival groups.

**Table 3:** Comparisons of arterial blood gas readings and ventilator-associated parameters of subjects stratified by survival status.

Categorical Variables	Survival Group (*n* = 29)	Non-Survival Group (*n* = 27)	*p*
pH			
Mean of Days 0–2	7.37 ± 0.07	7.32 ± 0.07	<0.05
Mean of Days 3–5	7.44 ± 0.13	7.41 ± 0.09	0.17
Mean of Days 6–7	7.43 ± 0.22	7.45 ± 0.22	0.88
PaO_2_ (mmHg)			
Mean of Days 0–2	152.24 ± 66.6	132.51 ± 39.59	0.29
Mean of Days 3–5	130.32 ± 55.56	109.95 ± 37.91	0.31
Mean of Days 6–7	126.11 ± 54.51	105.18 ± 42.39	0.12
PaCO_2_ (mmHg)			
Mean of Days 0–2	41.2 ± 9.0	45.25 ± 9.16	0.06
Mean of Days 3–5	42.12 ± 8.43	45.41 ± 10.24	0.13
Mean of Days 6–7	43.15 ± 7.44	47.12 ± 9.49	0.09
HCO_3_^−^ (mEq/L)			
Mean of Days 0–2	23.01 ± 3.77	22.53 ± 3.49	0.62
Mean of Days 3–5	25.77 ± 4.42	25.18 ± 3.94	0.59
Mean of Days 6–7	27.0 ± 6.79	27.04 ± 6.37	0.74
P/F ratio			
Mean of Days 0–2	245.09 ± 117.36	201.98 ± 79.74	0.21
Mean of Days 3–5	284.5 ± 165.67	213.48 ± 95.2	0.31
Mean of Days 6–7	298.97 ± 158.06	190.82 ± 93.9	<0.05
Mean airway pressure (mmHg)			
Mean of Days 0–2	16.65 ± 3.82	17.96 ± 3.11	0.25
Mean of Days 3–5	16.71 ± 4.45	17.81 ± 4.39	0.42
Mean of Days 6–7	16.08 ± 4.01	18.14 ± 4.31	0.07

Abbreviations: PH, potential of hydrogen; PaO_2_, partial pressure of oxygen in arterial blood; PaCO_2_, partial pressure of carbon dioxide in arterial blood; HCO_3_^−^, bicarbonate; P/F ratio, the ratio of arterial oxygen partial pressure to fractional inspired oxygen. Data are expressed as the mean ± standard deviation. Differences between groups were determined by Mann–Whitney U-test. The *p*-values were measured by comparing the survival and non-survival groups.

**Table 4:** Associations (odd ratios, ORs) of biochemistry measurements between the survival (with successful weaning) and non-survival groups.

Categorical Variables	Crude OR (95% CI) ^a^	Adjusted OR (95% CI) ^b^
CRP (mg/L)		
Mean of Days 0–2	1.1 (1.02 to 1.21) *	1.11 (1.01 to 1.22) *
Mean of Days 3–5	1.04 (0.95 to 1.14)	1.09 (0.98 to 1.22)
Mean of Days 6–7	0.94 (0.85 to 1.05)	0.96 (0.86 to 1.08)
Ferritin (ng/mL)		
Mean of Days 0–2	0.57 (0.09 to 3.67)	0.53 (0.06 to 4.83)
Mean of Days 3–5	0.56 (0.09 to 3.65)	0.49 (0.04 to 6.25)
Mean of Days 6–7	0.68 (0.32 to 1.43)	0.71 (0.33 to 1.51)
WBC (10^3^/μL)		
Mean of Days 0–2	0.96 (0.86 to 1.06)	0.97 (0.86 to 1.08)
Mean of Days 3–5	0.89 (0.8 to 1.0)	0.89 (0.79 to 1.01)
Mean of Days 6–7	0.89 (0.8 to 1.0)	0.88 (0.78 to 0.99)

Abbreviations: CRP, C-reactive protein; WBC, White blood cell count. ^a^ Simple logistic regression models. ^b^ Multivariable logistic regression models adjusted for age, sex, body mass index, and Charlson comorbidity index. * *p*-values < 0.05.

**Table 5:** Associations (odd ratios, ORs) of arterial blood gas readings and ventilator-associated parameters between the survival and non-survival groups.

Categorical Variables	Crude OR (95% CI) ^a^	Adjusted OR (95% CI) ^b^
PH		
Mean of Days 0–2	2.08 (1.11 to 3.89) *	2.22 (1.08 to 4.59) *
Mean of Days 3–5	1.32 (0.76 to 2.28)	1.11 (0.58 to 2.11)
Mean of Days 6–7	0.96 (0.57 to 1.62)	1.14 (0.62 to 2.11)
PaO_2_ (mmHg)		
Mean of Days 0–2	1.48 (0.82 to 2.7)	1.8 (0.91 to 3.55)
Mean of Days 3–5	1.56 (0.89 to 2.76)	2.32 (1.11 to 4.86) *
Mean of Days 6–7	1.61 (0.88 to 2.93)	2.11 (1.06 to 4.21) *
PaCO_2_ (mmHg)		
Mean of Days 0–2	0.95 (0.9 to 1.01)	0.91 (0.84 to 0.99) *
Mean of Days 3–5	0.96 (0.91 to 1.02)	0.95 (0.89 to 1.02)
Mean of Days 6–7	0.94 (0.88 to 1.01)	0.92 (0.85 to 0.99) *
HCO_3_^−^ (mEq/L)		
Mean of Days 0–2	1.04 (0.9 to 1.2)	0.98 (0.82 to 1.16)
Mean of Days 3–5	1.04 (0.91 to 1.18)	0.99 (0.85 to 1.15)
Mean of Days 6–7	1.01 (0.92 to 1.08)	0.98 (0.89 to 1.07)
P/F ratio		
Mean of Days 0–2	1.59 (0.88 to 2.88)	2.24 (1.02 to 4.93) *
Mean of Days 3–5	1.79 (0.95 to 3.36)	2.91 (1.23 to 6.89) *
Mean of Days 6–7	2.84 (1.29 to 6.23) *	3.84 (1.54 to 9.53) *
Mean airway pressure (mmHg)		
Mean of Days 0–2	0.68 (0.41 to 1.17)	0.49 (0.24 to 0.99) *
Mean of Days 3–5	0.78 (0.46 to 1.32)	0.71 (0.36 to 1.33)
Mean of Days 6–7	0.61 (0.35 to 1.05)	0.53 (0.26 to 1.07)

Abbreviations: PH, potential of hydrogen; PaO_2_, partial pressure of oxygen in arterial blood; PaCO_2_, partial pressure of carbon dioxide in arterial blood; HCO_3_^−^, bicarbonate; P/F ratio, the ratio of arterial oxygen partial pressure to fractional inspired oxygen. ^a^ Simple logistic regression models. ^b^ Multivariable logistic regression models adjusted for age, sex, body mass index, and Charlson comorbidity index. * *p*-values < 0.05.

## Data Availability

The datasets generated during and/or analyzed in the current study, along with the raw data supporting the conclusions, are available from the corresponding author upon reasonable request, as detailed in the article.

## Supplementary Materials

The following supporting information can be downloaded at: https://www.mdpi.com/article/10.3390/jcm13206190/s1, Figure S1. Bar chart assessing the importance of established models for predicting survival (with successful weaning) or non-survival with the mean of Days 0–5 and Days 0–7; Figure S2. Comparisons of performance between the model trained with the mean of Days 0–2, Days 0–5, and Days 0–7. Table S1: Comparisons of biochemistry measurements at various time lengths in subjects stratified by survival status; Table S2: Comparisons of arterial blood gas readings and ventilator-associated parameters at various time lengths in subjects stratified by survival status.
