# Supplementary material for: Predicting Survival Status in COVID-19 Patients: Machine Learning Models Development with Ventilator-Related and Biochemical Parameters from Early Stages: A Pilot Study
Source: J Clin Med. 2024 Oct 17;13(20):6190. doi: 10.3390/jcm13206190 (PMC11508203; doi:10.3390/jcm13206190)

Supplementary Materials

Table S1. Comparisons of biochemistry measurements at various time lengths in subjects stratified by survival status.

Categorical Variables	Survival group (n=29)	Non-survival group (n=27)	P
CRP (mg/L)			
Mean of Days 0-2	12.62 ± 8.15	7.51 ± 6.34	<0.05
Mean of Days 0-5	9.34 ± 5.85	6.01 ± 4.89	<0.05
Mean of Days 0-7	7.82 ± 4.51	5.74 ± 4.72	0.06
Ferritin (ng/mL)			
Mean of Days 0-2	1370.4 ± 864.34	3701.1 ± 12137.88	0.39
Mean of Days 0-5	1166.3 ± 661.79	3061.01 ± 9832.2	0.71
Mean of Days 0-7	1070.97 ± 589.9	2609.31 ± 7846.94	0.63
WBC (10^3/μL)			
Mean of Days 0-2	10.24 ± 5.47	11.39 ± 4.9	0.34
Mean of Days 0-5	10.23 ± 4.81	12.27 ± 4.48	0.11
Mean of Days 0-7	10.31 ± 4.56	12.61 ± 4.29	0.06

Abbreviations: CRP, C-reactive protein; WBC, White blood cell count. Data are expressed as the mean ± standard deviation. Differences between groups were determined by Mann-Whitney u-test. p values were measured by comparing between the survival and non-survival groups.

Table S2: Comparisons of arterial blood gas readings and ventilator-associated parameters at various time lengths in subjects stratified by survival status.

Categorical Variables	Survival group (n=29)	Non-survival group (n=27)	P
PH			
Mean of Days 0-2	7.37 ± 0.07	7.32 ± 0.07	<0.05
Mean of Days 0-5	7.39 ± 0.06	7.35 ± 0.06	<0.05
Mean of Days 0-7	7.39 ± 0.05	7.36 ± 0.06	<0.05
PaO_2 (mmHg)			
Mean of Days 0-2	152.24 ± 66.6	132.51 ± 39.59	0.29
Mean of Days 0-5	141.28 ± 55.5	121.23 ± 31.29	0.35
Mean of Days 0-7	137.49 ± 52.97	117.22 ± 31.79	0.21
$PaCO_2$ (mmHg)			
Mean of Days 0-2	41.2 ± 9.0	45.25 ± 9.16	0.06
Mean of Days 0-5	41.66 ± 7.69	45.33 ± 9.0	0.11
Mean of Days 0-7	42.03 ± 7.16	45.78 ± 8.54	0.08
HCO_3^- (mEq/L)			
Mean of Days 0-2	23.01 ± 3.77	22.53 ± 3.49	0.62
Mean of Days 0-5	24.39 ± 3.91	23.85 ± 3.49	0.59
Mean of Days 0-7	25.04 ± 4.14	24.65 ± 3.86	0.72
P/F ratio			
Mean of Days 0-2	245.09 ± 117.36	201.98 ± 79.74	0.21
Mean of Days 0-5	264.79 ± 133.95	207.73 ± 80.76	0.23
Mean of Days 0-7	273.34 ± 136.68	203.5 ± 78.07	<0.05
Mean airway pressure (mmHg)			
Mean of Days 0-2	16.65 ± 3.82	17.96 ± 3.11	0.25
Mean of Days 0-5	16.68 ± 3.75	17.88 ± 3.43	0.22
Mean of Days 0-7	16.53 ± 3.56	17.95 ± 3.5	0.14

Abbreviations: PH, potential of hydrogen; PaO_2, partial pressure of oxygen in arterial blood; $PaCO_2$, partial pressure of carbon dioxide in arterial blood; HCO_3^-, bicarbonate; P/F ratio, the ratio of arterial oxygen partial pressure to fractional inspired oxygen. Data are expressed as the mean ± standard deviation. Differences between groups were determined by Mann-Whitney u-test. p values were measured by comparing between the survival and non-survival groups.

Figure S1. Bar chart assessing the importance of established models for predicting survival (with successful weaning) or non-survival with the mean of Days 0-5 and Days 0-7

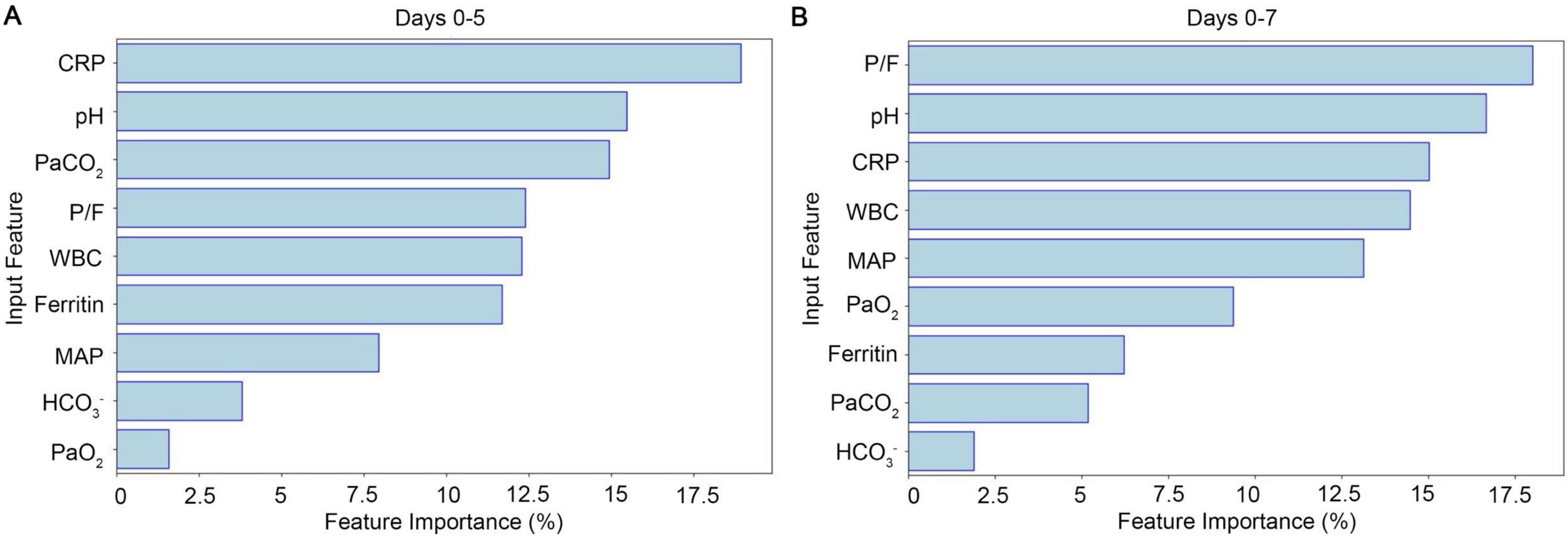

Figure S2. Comparisons of performance between the model trained with the mean of Days 0-2, Days 0-5, and Days 0-7

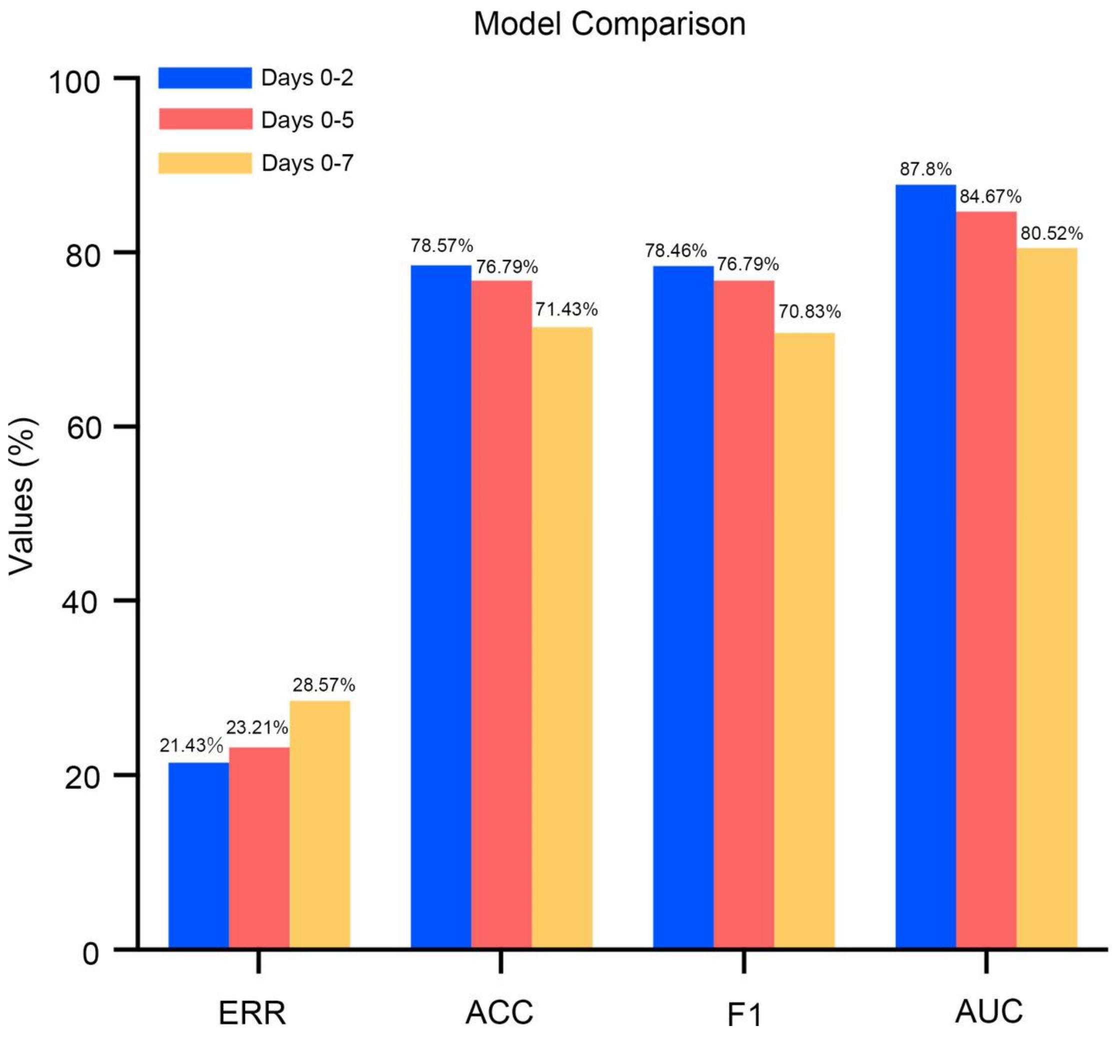

Supplement: Supplementary file 1 [file jcm-13-06190-s001.zip › jcm-3215858-supplementary.pdf]
